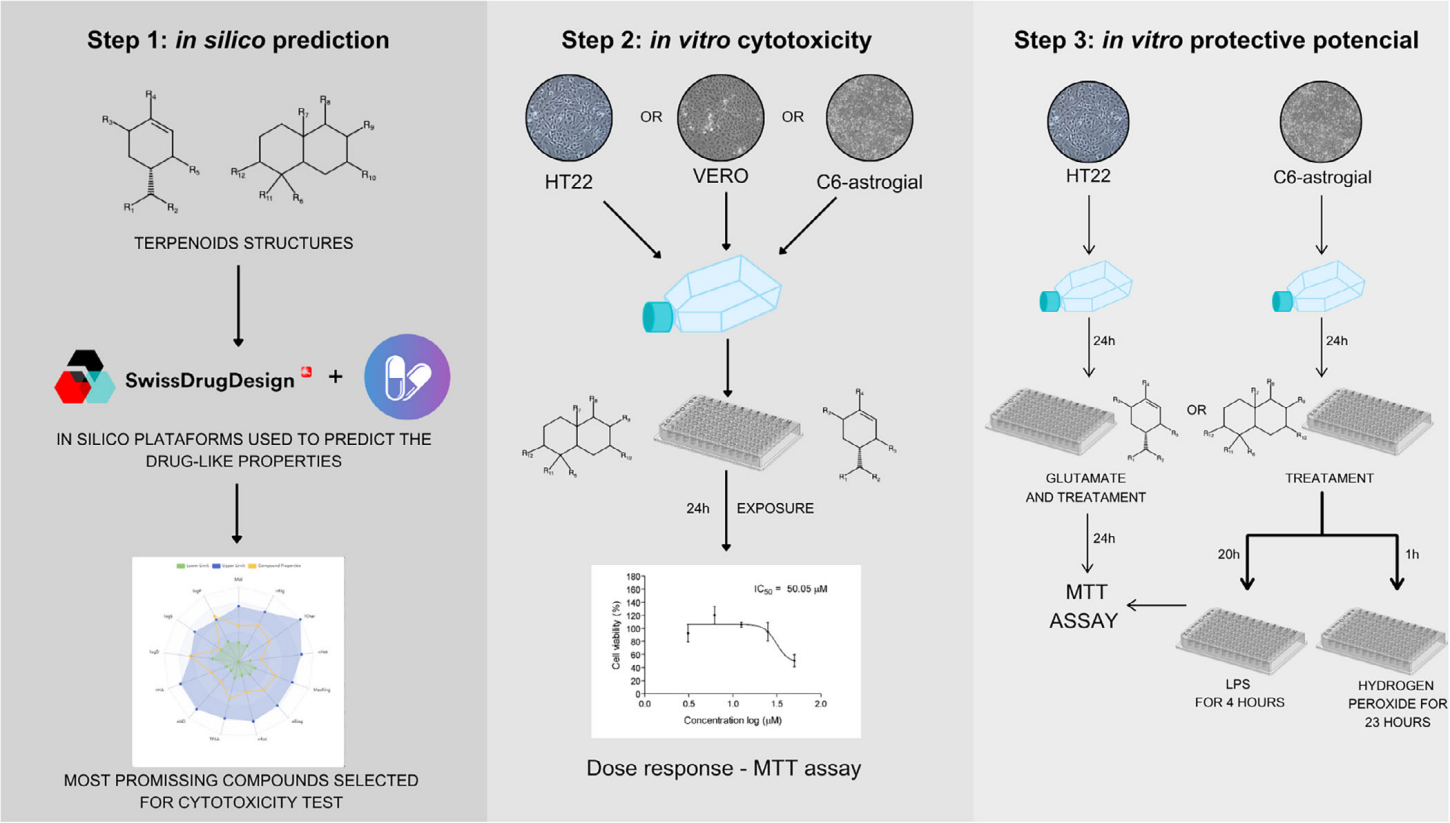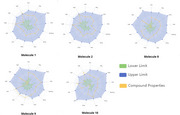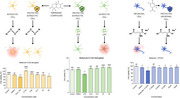# Terpenoid Derivatives from Asteraceae: A Multifactorial Path to Promising Multitarget Alzheimer's Therapy

**DOI:** 10.1002/alz70859_104674

**Published:** 2025-12-25

**Authors:** Murillo Orsatto Haas, Francielli Alana Pereira, Drielli Rhiane Peres Colhado Arêas, Mariana Koetz, Thaís Carine Ruaro, Maria Helena Sarragioto, José Ângelo Zuanazzi, Debora Cristina Baldoqui, Aline R. Zimmer

**Affiliations:** ^1^ Federal University of Rio Grande do Sul, Porto Alegre, Rio Grande do Sul Brazil; ^2^ State University of Maringá, Maringá, Paraná Brazil

## Abstract

**Background:**

Alzheimer's disease is a progressive neurodegenerative disorder and the leading cause of dementia worldwide. Its multifactorial nature involves damage to various structures and cell types of the central nervous system (CNS), submitted to multiple insults acting synergically, culminating in cognitive decline. This complexity inspired the search for new natural‐based molecules aiming for multitarget therapy for different CNS cells and structures.

**Method:**

Terpenoid derivatives isolated from different species of the Asteraceae family were evaluated in silico using SwissADME and ADMETLab 3.0 platforms. Most promising compounds were assayed for cytotoxicity using VERO (renal), HT22 (hippocampal neurons), and C6‐astroglial (astrocytic) cell lines. Cytoprotective capacities were evaluated against inflammatory (LPS‐induced), oxidative (H₂O₂‐induced), and glutamatergic (L‐glutamic acid‐induced) insults in HT22 and C6‐astroglial cell lines.

**Result:**

In silico predictions showed that 2 out of 5 compounds have potential permeability through the blood‐brain barrier (BBB), 3 out of 5 demonstrated high potential for gastrointestinal absorption, and 2 out of 5 were likely P‐glycoprotein substrates. Cytotoxicity tests revealed that all compounds exhibited IC₅₀ values between 50 and 100 µM in VERO, C6‐astroglial, and HT22 cell lines. Regarding C6‐astroglial cells cytoprotecting assays, 04 compounds demonstrated significant protection against inflammatory insults at 6.25 µM and against oxidative insult at 3.125 µM. In HT22 cell assays, all compounds offered substantial protection against glutamatergic excitotoxicity at 3.125 µM.

**Conclusion:**

The in silico evaluation showed satisfactory drug‐like properties for the terpenoid derivatives, including potential BBB permeability and high oral bioavailability. The cytotoxicity assays indicated low toxicity, with IC₅₀ values higher than 50 µM in all cell lines. The compounds displayed effective cytoprotective effects against oxidative, inflammatory, and glutamatergic insults in CNS‐relevant cell lines, emphasizing their promising potential for multitarget therapy in Alzheimer's disease. Notably, neuroprotective effects were observed in concentrations 16 times lower than cytotoxic ones, indicating a large therapeutic window. These findings suggest that the compounds could provide a solid foundation for further therapeutic development.